# Purification and Characterization of an Alkaline Lipase from *Streptomyces* sp. AU-153 and Evaluation of Its Detergent Compatibility

**DOI:** 10.1021/acsomega.5c11317

**Published:** 2026-01-23

**Authors:** Rukiye Boran Gulen, Aysel Ugur, Nurdan Sarac

**Affiliations:** † Medical Laboratory Program, Department of Medical Services and Techniques, Vocational School of Health Service, 220089 Aksaray University, 68100 Aksaray, Turkey; ‡ Section of Medical Microbiology, Department of Basic Sciences, Faculty of Dentistry, 37511 Gazi University, 06500 Ankara, Turkey; § Department of Biology, Faculty of Science, 52986 Mugla Sitki Kocman University, 48000 Mugla, Turkey

## Abstract

Detergent-compatible lipases are increasingly valued for their ability to remove stains under low-temperature and environmentally friendly washing conditions. Their industrial applicability depends on achieving high enzyme production, cost-effective purification, and stability within detergent formulations. Here, we report the purification and characterization of a highly active extracellular lipase from *Streptomyces* sp. AU-153 (1543 U/mL, *p*-NPP assay). A simplified aqueous two-phase system (ATPS) of poly­(ethylene glycol) and sodium chloride achieved 8-fold purification with a recovery of 272.7%. The purified enzyme exhibited optimal activity at pH 8.0 and 40 °C, maintained stability across pH 7–11, and retained substantial activity up to 60 °C. Activity was enhanced by Ca^2+^, Mg^2+^, and β-mercaptoethanol, whereas PMSF inhibited activity. The lipase remained stable in various commercial detergents and in the presence of surfactants, oxidizing agents, and boron compounds. It also showed affinity toward sunflower and thermally degraded olive oils. Low-temperature washing assays confirmed its effectiveness in oil stain removal. To our knowledge, ATPS-based purification and washing performance of *Streptomyces* lipases have each been reported only once, and this study is the first to integrate both approaches for the same enzyme. Moreover, *Streptomyces* sp. AU-153 displayed one of the highest native extracellular lipase activities documented for the genus, while the ATPS protocol achieved one of the highest recoveries reported for microbial lipases. These findings establish strain AU-153 as a promising natural source of detergent-compatible lipases and highlight its potential for enzyme-based washing applications.

## Graphical abstract

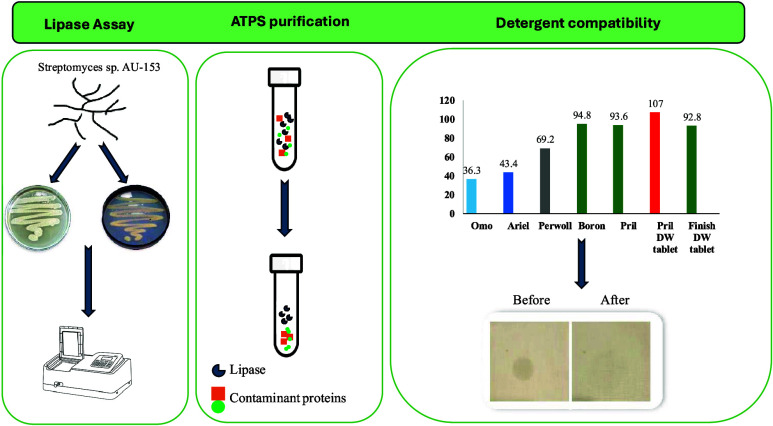

## Introduction

Enzymes have become pivotal components of green cleaning technologies, operating as environmentally sustainable alternatives to conventional surfactants. Proteases, lipases, amylases and cellulases enhance cleaning efficiency by selectively degrading protein-, fat-, starch- and cellulose-based stains under mild washing conditions. This process has been shown to minimize fabric damage, reduce reliance on harsh chemicals, and enable lower water and energy consumption.^1,2^ The utilization of these materials confers several advantages, including their biodegradability, nontoxicity, and operational stability during laundering. This approach is conducive to the development of more sustainable and energy-efficient washing processes.^1–4^ However, oxidizing agents, surfactants, and elevated wash temperatures, which are commonly used in detergents, have been shown to compromise enzyme stability and activity, thus limiting their industrial applicability.^5,6^ The development of robust, detergent-tolerant enzymes is therefore a key challenge in the field of modern detergent biotechnology.

Lipases (EC 3.1.1.3) are of particular importance in detergent formulations due to their ability to hydrolyze triglycerides present in oily stains. This reaction releases glycerol and free fatty acids, which can be more readily emulsified by surfactants, thereby enhancing grease removal efficiency at lower washing temperatures and reducing overall energy consumption.^1,4,7^ Industrial demand for microbial lipases is steadily increasing, with detergent applications representing one of the largest and fastest-growing market segments.^8^ For successful application in detergents, lipases must tolerate alkaline pH, surfactants, oxidizing agents, and other formulation additives.^1^ Although commercial lipases have been engineered for improved stability,^9^ they often require stabilizing compounds to maintain activity during storage and laundering.^10,11^ The identification of lipases that exhibit inherent robustness without extensive formulation support therefore remains a key objective in detergent biotechnology.

The large-scale recovery of catalytically active lipases remains a major challenge in industrial enzyme production. Conventional purification techniques, including chromatography and ultrafiltration, are often associated with high operational costs, extended processing times, and significant activity losses. These limitations have driven increasing interest in alternative purification strategies that are both cost-effective and enzyme-friendly.^12^ Aqueous two-phase systems (ATPS) have emerged as an attractive alternative, offering mild processing conditions, scalability, and high enzyme recovery while preserving catalytic activity.^13,14^ Despite these advantages, ATPS has been applied to *Streptomyces*-derived lipases only once, yielding moderate purification efficiency and recovery.^15^ This limited application highlights a clear opportunity to further explore ATPS-based purification strategies for *Streptomyces* lipases.

*Streptomyces* spp. represent a significant microbial reservoir for industrial enzymes, owing to their metabolic diversity and ecological versatility. They produce nearly 75% of all naturally occurring antibiotics and numerous bioactive metabolites.^16,17^ They also contribute significantly to biocatalysis, bioremediation, energy production, agriculture, food processing, and cosmetics.^18,19^ Their inherent ability to secrete large amounts of extracellular hydrolases and the presence of multiple lipase-encoding genes suggest considerable but underexplored enzymatic potential.^20^ Recent evaluations have identified *Streptomyces* as a novel and promising source of enzymes with broad biotechnological applicability.^17,21^

Despite these advantages, *Streptomyces* remain underrepresented in industrial enzyme production. Fungi account for 60% of commercial enzyme synthesis, bacteria for 24%, higher animals for 6%, yeast for 4%, and plants for 4%, whereas *Streptomyces* contribute only 2%.^22^ This underutilization is likely due to slower growth rates, complex metabolism, and the historical focus on well-characterized bacterial lipases. Consequently, relatively few *Streptomyces* lipases have been purified and biochemically characterized, with studies evaluating detergent compatibility being particularly scarce. To date, only a single report has assessed the washing performance of a *Streptomyces*-derived lipase,^23^ despite the organism’s nonpathogenic nature, ability to grow on inexpensive media, and strong extracellular enzyme secretion. This scarcity underscores a clear research gap in the identification and development of detergent-compatible *Streptomyces* lipases.

To address this gap, the present study investigates a *Streptomyces*-derived lipase with potential applicability in laundry detergent formulations. The *Streptomyces* sp. AU-153 strain used in this study was isolated from soil in Mugla Province, Turkey^24^ and selected for its high extracellular lipase activity.^25^ The objectives were to evaluate lipase production by the strain, purify the enzyme using an ATPS to preserve catalytic activity, characterize its biochemical properties, and assess both its compatibility with detergent ingredients and its practical performance in oily stain removal tests. To our knowledge, this represents only the second study applying ATPS to *Streptomyces* lipase purification and the second evaluating its washing efficacy, providing new insights into environmentally friendly, robust, and industrially relevant biocatalysts.

## Materials and Methods

### Materials

The chemicals and reagents used for enzyme purification, characterization, and activity assays—including substrates and buffers—were of analytical grade and were obtained from Sigma-Aldrich and Merck.

### Bacteria and Lipase Activity Screening

*Streptomyces* sp. AU-153 was previously isolated from soil samples collected in Mugla Province, Turkey,^24^ and subsequently deposited in the Mugla Sitki Kocman University Culture Collection. In a later investigation, AU-153 and other *Streptomyces* isolates in the collection were screened for extracellular lipase production.^25^ The screening indicated that AU-153 possessed one of the highest extracellular lipase activities, which justified its selection for detailed characterization.

The preliminary lipase activity was assessed on two indicator media: Tributyrin Agar and Rhodamine B Agar.^25^ Following this screening step, quantitative enzyme production was carried out using International *Streptomyces* Project 2 (ISP2) broth medium. For this purpose, the isolate was cultured on ISP2 agar plates at 30 °C for 7 days to obtain spores, which were harvested using 0.01% (v/v) Tween 80. A 2% (v/v) spore suspension was then used to inoculate ISP2 broth, followed by incubation at 30 °C for 7 days.^25^ After incubation, cultures were centrifuged at 10,000*g* for 15 min at 4 °C, and the supernatant was collected as the source of extracellular lipase.

Lipase activity was quantified spectrophotometrically using *p*-nitrophenyl palmitate (*p*-NPP) as the substrate, following modified protocols of Winkler and Stuckmann^26^ and Boran and Uğur.^27^ One unit (U) of activity corresponded to the release of 1 μmol of *p*-nitrophenol per minute. The reaction mixture contained 1 mL enzyme solution and 9 mL substrate solution (30 mg p-NPP dissolved in 10 mL isopropanol, added to 90 mL of 50 mM Tris-HCl pH 8.0, with 2 mL Triton X-100 and 0.1 g gum arabic). The mixture was incubated at 30 °C for 30 min. All assays were performed in triplicate, and absorbance was measured at 410 nm. Activity was calculated using

1 $$l\mathrm{ipase activity}\;\left(U/\mathrm{mL}\right)=\frac{\left(A_{410}\times V_{\mathrm{total}}\times \mathrm{dilution factor}\right)}{t\times V_{\mathrm{enzyme}}}$$

where: *A*_410_ = absorbance at 410 nm, *V*_total_ = total reaction volume (mL), *V*_enzyme_ = enzyme sample volume (mL), *t* = incubation time (min)

### Lipase Purification Using Aqueous Two-Phase System

The extracellular lipase was purified using an ATPS composed of PEG 4000 (12% w/w), NaCl (2% w/w), and a K_2_HPO_4_/KH_2_PO_4_ buffer system (13% w/w, pH 7.0).^15^ An appropriate amount of crude enzyme was introduced to achieve a final concentration of 20% (w/w), and the system mass was adjusted to 10 g with distilled water. Following vortex mixing for 5 min, the mixture was maintained at 25 °C for 24 h under static conditions to promote phase formation.

Phase clarification was performed by centrifugation at 4000*g* for 10 min at 4 °C. Two phases were recovered: a PEG-rich upper phase and a salt-rich lower phase. Both phases became clear and transparent, and the interface was well-defined. Phases were separated carefully to prevent mixing, and their volumes were recorded. Samples from each phase were subjected to protein quantification and lipase activity assays.

The partition coefficient (*K*_e_), purification fold (PF), and enzyme recovery (*Y*) were calculated as described by Alhelli et al.^28^

The *K*_e_ value was calculated from the activity measured in the top (*A*_*t*_) and bottom phases (*A*_*b*_).

2 $$K_{e}=\frac{A_{t}}{A_{b}}$$

The purification fold in the top phase (*P*_FT_) and bottom-phase (*P*_FB_) was calculated by comparing the specific activity in each phase (*S*_AT_ and *S*_AB_, respectively) with that of the crude extract (SA_CE_).

3 $$P_{FT}=\frac{S_{AT}}{SA_{CE}}$$

4 $$P_{\mathrm{FB}}=\frac{S_{\mathrm{AB}}}{SA_{\mathrm{CE}}}$$

Enzyme recovery in the top phase (*Y*_T_) or bottom-phase (*Y*_B_) was calculated as the percentage of total activity in the top (*A*_T_) or bottom (*A*_B_) phase relative to the initial activity in the crude extract (*A*_C_).

5 $$Y_{T}\left(\%\right)=\frac{A_{T}}{A_{c}}\times 100$$

6 $$Y_{B}\left(\%\right)=\frac{A_{B}}{A_{c}}\times 100$$

### Effect of pH and Temperature on Lipase Activity and Stability

The pH profile of the lipase was evaluated using *p*-NPP in 500 mM buffer systems: citrate–phosphate (pH 6.0), Tris–HCl (pH 7.0–9.0), and glycine–NaOH (pH 10.0–11.0). The optimum pH was 8.0, which was used in subsequent assays. pH stability was assessed by incubating the enzyme in the respective buffers at 25 °C for 1 h. An enzyme sample maintained in buffer at pH 8.0 without any preincubation served as the control, and its activity was defined as 100%.

The temperature profile was evaluated between 30 °C and 60 °C at the optimum pH. The optimum temperature was 40 °C and was used for all further experiments. Thermal stability was assessed by incubating the enzyme at selected temperatures for 1 h. An enzyme sample maintained at 25 °C without prior incubation served as the control and was assigned an activity value of 100%.

### Effects of Inhibitors, Metal Ions, and Boron Compounds on Lipase Stability

Enzyme stability was evaluated by exposing the lipase to various additives at 25 °C for 1 h. The tested compounds comprised inhibitors (EDTA, PMSF, iodoacetic acid, β-mercaptoethanol; 0.1% w/v), metal ions (Ca^2+^, Mg^2+^, Mn^2+^, Fe^2+^, Cu^2+^, Co^2+^, K^+^, Zn^2+^; 5 mM), and boron-based compounds (potassium metaborate, boric acid, sodium metaborate, sodium tetraborate; 5 mM). Residual activity was expressed relative to the untreated control (100%).

### Effects of Detergent Additives on Lipase Stability

To examine the influence of detergent-related additives on lipase stability, the enzyme was exposed at 25 °C for 1 h to selected nonionic and anionic surfactants, including Tween 40, Tween 60, Tween 80, Triton X-100, SDS, sodium cholate, and saponin (each at 1% w/v). In addition, the effect of the antiredeposition agent sodium carbonate (10 mM) and common oxidizing agents, namely sodium hypochlorite, hydrogen peroxide, and sodium perborate (0.1% w/v), was evaluated. Following incubation, residual enzymatic activity was determined using the standard assay protocol and expressed as a percentage of the untreated control.

### Compatibility with Commercial Detergents

Compatibility was evaluated using commercially available detergent products, including powder detergents Omo (Unilever, Turkey), Ariel (Procter and Gamble, Belgium), and Boron (Eti Maden, Turkey); liquid detergents Perwoll (Henkel, Germany) and Pril (Henkel, Austria); and dishwasher tablet detergents Pril (Henkel, Austria) and Finish (Reckitt Benckiser, Poland). Each detergent was diluted to 1% (w/v) and heat-treated at 100 °C for 1 h to inactivate native enzymes. After cooling, the enzyme was incubated with each detergent solution for 1 h at 25 °C, and residual activity was measured relative to a detergent-free control.

### Substrate Specificity toward Natural Oils

Lipase hydrolytic activity toward various natural oils—including corn oil, sunflower oil, olive oil, soybean oil, and thermally degraded olive oil—was determined using a titrimetric assay described by Ugur and Boran.^29^ The highest activity observed among all substrates was taken as 100% for comparative evaluation.

### Washing Performance Evaluation

Washing performance was assessed using white cotton fabric cut into pieces of approximately 5 cm ×  5 cm, each stained with sunflower oil. Samples were subjected to four washing conditions: tap water only, tap water with lipase (25 U), tap water with heat-inactivated detergent (1% w/v), and tap water with heat-inactivated detergent (1% w/v) supplemented with lipase (25 U), following a modified protocol of Safdar et al.^3^ Each washing treatment was performed in a shaking incubator at 40 °C (optimal temperature) for 30 min at 120 rpm. After washing, the fabrics were rinsed with tap water and air-dried before weighing. Oil removal efficiency was calculated using the fabric weights before staining, after staining, and after washing as follows

7 $$\mathrm{oil}\;\mathrm{removal}\;\mathrm{efficiency}\left(\%\right)=\frac{W_{3}-W_{1}}{W_{2}-W_{1}}\times 100$$

where: *W*_1_ = weight of fabric before staining, *W*_2_ = weight after staining, *W*_3_ = weight after washing.

### Statistical Analysis

Experiments were independently repeated three times. Data are reported as mean values ± standard deviation. Group comparisons were performed using one-way ANOVA, and results with *p* values below 0.05 were considered statistically significant.

## Results and Discussion

### Lipase Activity Screening

The lipolytic potential of *Streptomyces* sp. AU-153 was initially verified by tributyrin agar screening, where a clear hydrolysis halo was observed around the colonies (Figure 1A). Lipolytic activity was further confirmed using the Rhodamine B agar assay, a sensitive method for detecting lipase-mediated hydrolysis of long-chain triglycerides. The presence of orange-red fluorescence under UV illumination was indicative of lipolytic activity in the isolate (Figure 1B).

**Figure 1:**
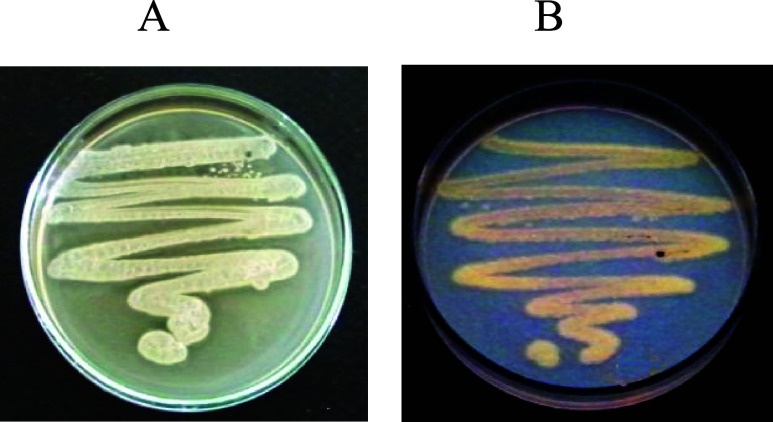
Lipase activity of *Streptomyces* sp. AU-153 on agar media: (A) tributyrin agar with clear hydrolysis halos and (B) Rhodamine B agar under UV illumination.

Quantitative analysis showed that *Streptomyces* sp. AU-153 produced 1543 U/mL of extracellular lipase after 7 days of incubation using p-nitrophenyl palmitate as the substrate. In contrast, most native *Streptomyces* strains reported in the literature exhibit extracellular lipase activities below 300 U/mL,^30,31^ with only a few isolates reaching 300–550 U/mL.^32–34^ To the best of our knowledge, extracellular lipase activities exceeding 1000 U/mL have not been reported for native *Streptomyces* isolates under nonoptimized conditions. These findings indicate that AU-153 possesses an intrinsically high lipolytic capacity, even in the absence of medium optimization or induction.

### Lipase Purification Using Aqueous Two-Phase System

Green bioprocessing strategies aim to reduce chemical consumption, energy input, and downstream processing complexity during enzyme purification. In this context, ATPS based on water-rich and nontoxic components have emerged as attractive alternatives to conventional chromatographic methods for enzyme recovery.^12,13^ The extracellular lipase from *Streptomyces* sp. AU-153 was efficiently purified using a PEG 4000/NaCl/potassium phosphate-based ATPS (Table 1). Under the selected conditions, the enzyme preferentially partitioned into the polymer-rich top phase, resulting in an 8.30-fold purification and an activity recovery of 272.74%. The specific activity increased markedly, while only a small fraction of the total activity remained in the bottom phase, indicating a strong affinity of the AU-153 lipase for the PEG-rich environment.

**Table 1:** Purification of the Lipase from *Streptomyces* sp. Strain AU-153

purification step	partition coefficient (*K*_e_)	total activity (units)	total protein (mg)	specific activity (U/mg)	yield (*Y*) (%)	purification fold (PF)
crude lipase extract		708.66	1.0252	691.24	100	1
top phase	17.15	1932.81	0.337	5735.36	272.74	8.3
bottom phase		154.6	1.102	140.25	10.644	0.055

Recoveries exceeding 100% have been frequently reported in ATPS-based enzyme purification and are commonly attributed to phase-induced activation effects and the removal of inhibitory contaminants.^35–38^ In the present study, the unusually high recovery is most likely associated with interfacial activation combined with preferential partitioning of nonenzymatic components into the salt-rich phase, resulting in enhanced measurable activity.^35,38,39^ Similar phenomena have been documented for other microbial enzymes purified using ATPS.^38,40^ To the best of our knowledge, ATPS-based purification of a *Streptomyces*-derived lipase has been reported only once previously, yielding a 68% recovery and a 7.08-fold purification for *Streptomyces cellulosae* AU-10.^15^ In this context, the recovery achieved for the AU-153 lipase ranks among the highest reported for native *Streptomyces* lipases purified using ATPS.

The selected ATPS enabled efficient single-step purification while enhancing enzymatic activity, supporting its potential as an industrially relevant strategy. The simplicity, scalability, and environmental compatibility of this process align with current green bioprocessing principles aimed at reducing chemical usage, energy demand, and downstream complexity.^12,13,41^ Taken together with the intrinsically high production level of *Streptomyces* sp. AU-153, these features highlight this lipase as a promising candidate for sustainable detergent enzyme applications.

### Effect of pH and Temperature on Lipase Activity and Stability

The lipase produced by *Streptomyces* sp. AU-153 exhibited maximal activity at pH 8.0 and retained substantial activity across the alkaline range of pH 8.0–11.0 (Figure 2A). Stability assays showed full catalytic activity after incubation between pH 7.0 and 11.0, indicating strong resistance to alkaline denaturation (Figure 2B). Similar alkaline stability profiles have been reported for lipases from other *Streptomyces* strains, including *Streptomyces* sp. AU-1, *S. cellulosae* AU-10, *Streptomyces violascens* OC125–8, and *Streptomyces gobitricini*.^15,34,42,43^

**Figure 2:**
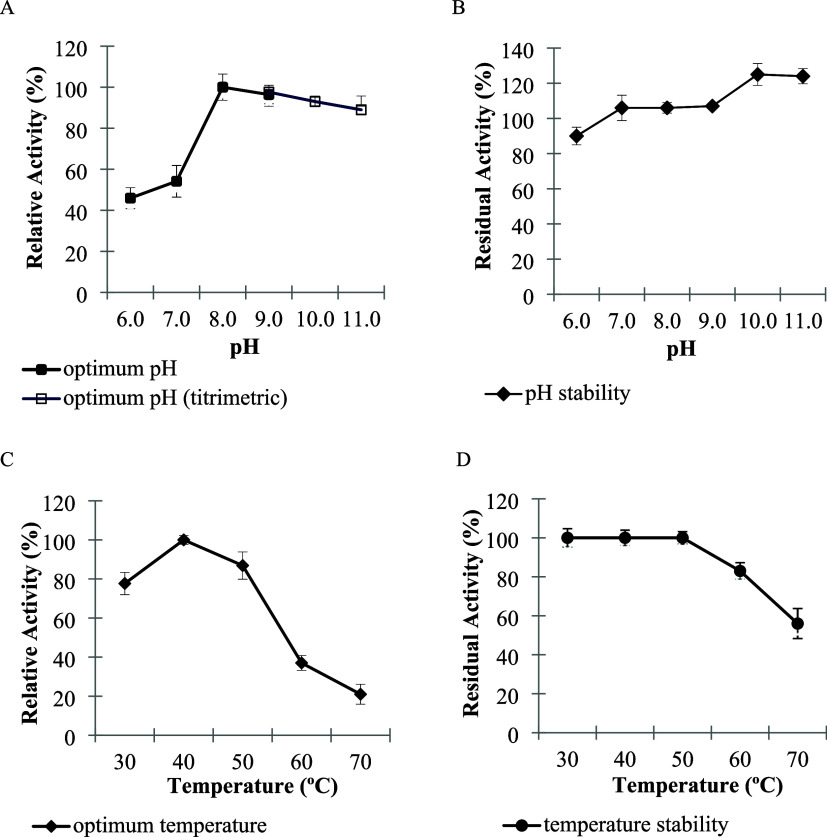
Effect of pH on lipase activity (A) and stability (B). Effect of temperature on lipase activity (C) and stability (D). All experiments were performed in triplicate, and results are expressed as mean ± SD (*n* = 3). Different letters indicate statistically significant differences at *p* < 0.05 (one-way ANOVA).

The enzyme exhibited optimal activity at 40 °C, retaining 77.67 ± 5.7% and 86.87 ± 7.0% activity at 30 and 50 °C, respectively (Figure 2C). Thermal stability assays demonstrated complete activity preservation between 30 and 60 °C, indicating pronounced thermostability (Figure 2D). Comparable temperature optima have been reported for other *Streptomyces*-derived lipases.^34,43,44^

Overall, the AU-153 lipase exhibits notable alkaline and thermal tolerance, satisfying key requirements for detergent enzymes operating under alkaline pH and variable washing temperatures.^4,5^

### Effects of Various Factors on Lipase Stability

Recent strategies to improve detergent enzyme performance have focused on protein engineering, chemical modification, and immobilization to enhance stability under harsh washing conditions. However, these approaches often increase cost and process complexity, limiting large-scale application.^5,9,41^ Consequently, enzymes that naturally tolerate alkaline pH, temperature fluctuations, and detergent ingredients are of particular interest.

### Effects of Inhibitors on Lipase Stability

To elucidate the biochemical nature of *Streptomyces* sp. AU-153 lipase, its activity was evaluated in the presence of various inhibitors and additives (Table 2). PMSF caused the most pronounced reduction in activity (43.7 ± 0.47%, *p* < 0.05), indicating the involvement of a serine residue in catalysis. Iodoacetic acid had no effect on activity (100 ± 2.5%), suggesting that cysteine residues are not essential. EDTA caused only a modest reduction in activity (91.3 ± 1.06%), indicating that the enzyme is not strictly metal-dependent and is tolerant to chelating agents commonly used in detergent formulations.^45^ β-Mercaptoethanol significantly enhanced lipase activity (127.9 ± 2.1%, *p* < 0.05), consistent with reports for other microbial lipases and suggesting limited involvement of sulfhydryl groups in catalysis.^46^

**Table 2:** Effect of Various Inhibitors, Metal Ions, Boron Compounds, Surfactants, Oxidizing Agents, and Commercial Detergents on the Lipase Enzyme^a^

chemical agents	concentration	residual activity (%)
control	-	100 ± 0.02^a^
EDTA	0.1% (w/v)	98.3 ± 0.91^a^
PMSF	0.1% (w/v)	43.7 ± 0.47^c^
iodoacetic acid	0.1% (w/v)	100 ± 2.5^a^
β-mercaptoethanol	0.1% (w/v)	127.9 ± 2.1^d^
Ca^2+^	5 mM	107.3 ± 0.35^e^
Mg^2+^	5 mM	112 ± 0.29^e^
Mn^2+^	5 mM	102.7 ± 0.21^d^
Fe^2+^	5 mM	107.8 ± 0.52^e^
Cu^2+^	5 mM	108.5 ± 0.81^e^
Co^2+^	5 mM	102.3 ± 2.5^d^
K^+^	5 mM	109.9 ± 2.5^e^
Zn^2+^	5 mM	36 ± 2.2^f^
boric acid	1 mM	98.3 ± 0.91^a^
potassium metaborate	1 mM	100.0 ± 0.40^a^
sodium metaborate	1 mM	98.6 ± 0.75^a^
sodium tetraborate	1 mM	101.0 ± 2.5^a^
Tween 40	1% (w/v)	87.6 ± 1.0^b^
Tween 60	1% (w/v)	89.1 ± 2.5^b^
Tween 80	1% (w/v)	87.9 ± 0.97^b^
Triton X-100	1% (w/v)	100.0 ± 1.43^a^
SDS	1% (w/v)	47.7 ± 2.5^c^
sodium cholate	1% (w/v)	106.0 ± 2.5^d^
saponin	1% (w/v)	117.2 ± 1.9^d^
sodium carbonate	10 mM	84.9 ± 2.3^e^
Omo	1% (w/v)	36.3 ± 1.04^c^
Ariel	1% (w/v)	43.4 ± 2.5^c^
Perwoll	1% (w/v)	69.2 ± 2.5^b^
Boron	1% (w/v)	94.8 ± 1.11^a^
Pril	1% (w/v)	93.6 ± 0.54^a^
Pril dishwasher tablet	1% (w/v)	107 ± 0.21^d^
Finish dishwasher tablet	1% (w/v)	92.8 ± 2.1^a^

a Residual activity of the untreated enzyme was set as 100%. Data are mean ± SD (*n* = 3). Different letters in the same rectangle indicate significant differences at *p* < 0.05.

### Effects of Metal Ions on Lipase Stability

Metal ions are known to influence lipase activity and stability by stabilizing active conformations and improving substrate accessibility. In the present study, both divalent and monovalent cations, including Ca^2+^, Mg^2+^, Mn^2+^, Fe^2+^, Cu^2+^, Co^2+^, and K^+^, significantly enhanced or maintained lipase activity compared to the control (Table 2, *p* < 0.05). In contrast, Zn^2+^ caused a marked reduction in activity, indicating an inhibitory interaction. The stabilizing role of Ca^2+^ in preserving lipase activity and preventing thermal denaturation has been widely reported,^7,12,39^ and similar activating effects of Fe^2+^, Cu^2+^, and Mn^2+^ have been described for detergent-compatible lipases from other *Streptomyces* species.^15,43,47^

### Effects of Boron-Based Compounds on Lipase Stability

Boron-based compounds are widely used in detergent formulations due to their multifunctional roles, including water softening and pH buffering.^48,49^ AU-153 lipase retained nearly full activity in the presence of all tested boron compounds, with no significant differences compared to the control (Table 2, *p* ≥ 0.05). This behavior is consistent with previous reports on detergent-compatible enzymes and supports the suitability of AU-153 lipase for incorporation into boron-containing detergent formulations.^15,50^

### Effects of Detergent Additives on Lipase Stability

Surfactants are key components of detergent formulations and play a central role in stain solubilization and emulsification.^51^ Therefore, lipase stability in the presence of surfactants is a critical parameter for detergent compatibility. In this study, the lipase from *Streptomyces* sp. AU-153 exhibited high stability toward nonionic surfactants, retaining substantial residual activities in the presence of Tween 40 (87.6 ± 1.0%), Tween 60 (89.1 ± 2.5%), Tween 80 (87.9 ± 0.97%), and Triton X-100 (100.0 ± 1.43%) (Table 2, *p* < 0.05). This stability profile exceeds that reported for the commercial enzyme Lipolase, which shows reduced activity in the presence of Triton X-100 and Tween 80.^34^ Similar protective effects of nonionic surfactants have been reported for other *Streptomyces* lipases.^15,34^

In contrast, AU-153 lipase activity was markedly reduced by the anionic surfactant SDS (47.7 ± 2.5%, *p* < 0.05), consistent with its strong protein-denaturing effect. Sodium cholate slightly enhanced activity (106.0 ± 2.5%), indicating a surfactant structure–dependent response.

Regarding oxidizing agents, AU-153 lipase retained high residual activity in the presence of sodium hypochlorite (94.3 ± 0.90%) and sodium perborate (88.9 ± 1.34%), whereas hydrogen peroxide caused a pronounced activity loss (49.1 ± 1.12%) (Table 2, *p* < 0.05). This oxidative stability profile, comparable to that reported for Lipolase and other *Streptomyces* lipases, supports the suitability of AU-153 lipase for detergent formulations containing common oxidizing agents.^15^

### Compatibility with Commercial Detergents

The stability of enzymes in commercial detergent formulations is a critical determinant of their industrial applicability. Detergents vary widely in composition, particularly with respect to surfactant types, oxidizing agents, alkaline builders, and chelating compounds, all of which can differentially affect enzyme structure and activity.

In the present study, the AU-153 lipase exhibited variable stability profiles depending on detergent formulation (Table 2). While high residual activity was observed in several liquid and dishwasher detergents (Pril dishwasher tablet (107 ± 0.21%), Boron (94.8 ± 1.11%), Pril liquid (93.6 ± 0.54%), and Finish dishwasher tablet (92.8 ± 2.1%) (*p* < 0.05)), substantial activity loss occurred in certain powder detergents (Omo (36.3 ± 1.04%) and Ariel (43.4 ± 2.5%) (*p* < 0.05)). Such differences are commonly attributed to the more aggressive chemical composition of powder detergents, which often contain higher levels of strong anionic surfactants, bleaching agents, and oxidizing systems known to promote enzyme denaturation.^52,53^ These findings highlight the formulation-dependent nature of detergent compatibility and emphasize the importance of enzyme-specific stability assessments.

Comparable detergent compatibility profiles have been reported for other *Streptomyces* lipases, including *S. cellulosae* AU-10, *S. violascens* OC125–8, and *S. gobitricini*.^15,34,43^ As a reference enzyme, Lipolase retained full activity in several detergents but showed reduced stability in specific formulations under comparable conditions.^54^ Collectively, these findings indicate that detergent compatibility depends not only on detergent composition but also on the intrinsic structural robustness of individual lipases against oxidants, anionic surfactants, and alkaline builders.

### Substrate Specificity toward Natural Oils

The hydrolytic activity of *Streptomyces* sp. AU-153 lipase toward various edible oils was evaluated using a titrimetric assay (Figure 3). Sunflower oil was used as the reference substrate (100 ± 3.0%). All other oils exhibited significantly lower hydrolytic activities (*p* < 0.05). Soybean oil and thermally degraded olive oil showed residual activities of 51.22 ± 1.3% and 41.66 ± 2.7%, respectively, while corn oil and olive oil showed low hydrolytic activity (∼17% for both).

**Figure 3:**
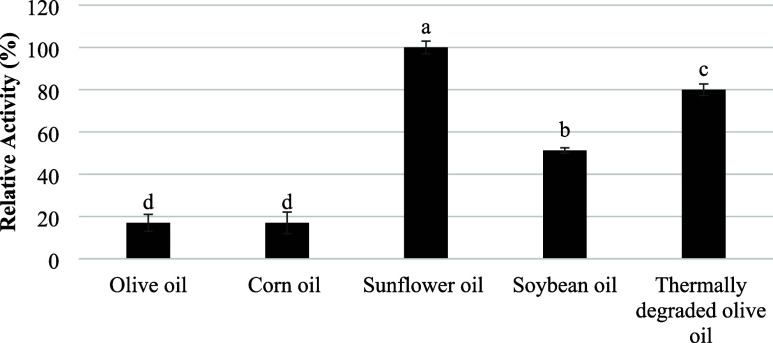
Substrate specificity of *Streptomyces* sp. AU-153 lipase toward different edible oils. Hydrolytic activity was determined by quantifying released free fatty acids using titration with 0.05 M NaOH and phenolphthalein as an indicator. Activities are expressed as relative activity, with sunflower oil used as the reference substrate (100%). Error bars represent the standard deviation of triplicate measurements (*n* = 3). Different letters indicate statistically significant differences (one-way ANOVA, *p* < 0.05).

This substrate preference profile is consistent with previous reports on *Streptomyces*-derived lipases. For example, the lipase from *S. cellulosae* AU-10 showed high activity toward sunflower oil (93.3%), with reduced hydrolysis of corn oil (60%) and thermally degraded olive oil (46.6%).^15^ Similarly, *S. violascens* OC125–8 lipase exhibited strong activity toward sunflower and olive oils, whereas oxidized or thermally altered oils were hydrolyzed less efficiently.^43^ These findings indicate that fatty acid composition, chain length distribution, and oxidative modifications influence substrate recognition and catalytic efficiency of *Streptomyces* lipases.

### Washing Performance Evaluation

The washing efficacy of *Streptomyces* sp. AU-153 lipase was evaluated by measuring sunflower oil removal from white cotton fabric under four washing conditions. The quantitative results are summarized in Table 3. Representative washing images corresponding to the experimental setup are provided in the Supporting Information (Figure S1).

**Table 3:** Effect of *Streptomyces* sp. AU-153 Lipase and Detergent on Sunflower Oil Removal from Cotton Fabric^a^

treatment condition	oil removal efficiency (%)
tap water only (control)	13.45 ± 0.05
tap water + lipase (25 U)	30.0 ± 1.0*
tap water + heat-inactivated detergent (1% (w/v))	31.12 ± 1.4*
tap water + heat-inactivated detergent (1% (w/v)) + Lipase (25 U)	32.3 ± 0.1*

a Sunflower oil-stained cotton fabrics were washed at 40 °C for 30 min under the indicated conditions. Data are presented as mean ± SD (*n* = 3). Asterisks (*) denote significant differences versus the control (*p* < 0.05, one-way ANOVA). No significant difference was observed between the combined treatment (lipase + detergent) and the individual detergent-only treatments (*p* > 0.05).

Lipase-only treatment significantly increased oil removal (30 ± 1.0%) compared to the water-only control (13.45 ± 0.05%) (*p* < 0.05). Washing with heat-inactivated detergent alone resulted in a comparable oil removal efficiency (31.12 ± 1.4%) (*p* < 0.05). The combined application of lipase and heat-inactivated detergent yielded a slightly higher oil removal efficiency (32.3 ± 0.1%), although this increase was not statistically significant compared with either treatment applied individually.

These results demonstrate that AU-153 lipase contributes to oil stain hydrolysis and enhances washing performance, consistent with recent studies reporting that detergent-compatible bacterial enzymes improve oil stain removal in mild, environmentally friendly, and enzyme-assisted laundering systems.^1,3,4,55^ In addition, the relatively low cost of using crude or partially purified enzyme further supports its practical and economic feasibility for detergent applications.^56^

However, studies specifically addressing the washing performance of *Streptomyces*-derived lipases remain limited. To date, only a single study has reported such an evaluation.^23^ The present study therefore provides additional experimental evidence supporting the application potential of *Streptomyces*-derived lipases in detergent formulations.

## Conclusions

In this study, *Streptomyces* sp. AU-153 was shown to produce a detergent-compatible extracellular lipase with high catalytic activity. The enzyme was efficiently purified using an ATPS, achieving high recovery while preserving enzymatic activity. The purified lipase exhibited stability over a broad alkaline pH range, moderate temperatures, and in the presence of metal ions, detergent additives, and commercial detergents, consistent with the operational requirements of modern laundry processes.

The AU-153 lipase contributed to oil stain removal from cotton fabrics under mild washing conditions, supporting its potential application in energy-efficient and environmentally friendly detergent formulations. Moreover, the successful integration of ATPS-based purification with functional washing performance evaluation highlights the suitability of this approach for sustainable enzyme production. Given the limited number of studies addressing detergent applications of *Streptomyces*-derived lipases, this work provides additional insight into their practical potential.

Overall, AU-153 lipase represents a promising candidate for detergent formulations and demonstrates the advantages of ATPS as a simple, scalable, and environmentally benign purification strategy for industrial enzyme development.

## Supplementary Material



## Notes

Photographic evidence of sunflower oil stain removal by the lipase produced by *Streptomyces* sp. AU-153. The figure shows oil-stained cloth samples before treatment and after treatment at 40 °C for 30 min with tap water, lipase alone, heat-inactivated detergent, and heat-inactivated detergent combined with the enzyme (Figure S1) (PDF)
