# Correction to “Purification and Characterization of an Alkaline Lipase from *Streptomyces* sp. AU-153 and Evaluation of Its Detergent Compatibility”

**DOI:** 10.1021/acsomega.6c01282

**Published:** 2026-05-29

**Authors:** Rukiye Boran Gulen, Aysel Ugur, Nurdan Sarac

In Table 2, the experimental results for sodium hypochlorite, hydrogen peroxide, and sodium perborate were inadvertently omitted in the published version of the article. The corrected table now includes these missing data.

**Table 2:** Effect of Various Inhibitors, Metal Ions, Boron Compounds, Surfactants, Oxidizing Agents, and Commercial Detergents on the Lipase Enzyme^a^

**Chemical agents**	**Concentration**	**Residual activity (%)**
**Control**		100 ± 0.02^a^
**EDTA**	0.1% (w/v)	91.3 ± 1.06^b^
**PMSF**	0.1% (w/v)	43.7 ± 0.47^c^
**Iodoacetic acid**	0.1% (w/v)	100 ± 2.5^a^
**β-Mercaptoethanol**	0.1% (w/v)	127.9 ± 2.1^d^
**Ca^2+^**	5 mM	107.3 ± 0.35^e^
**Mg^2+^**	5 mM	112 ± 0.29^e^
**Mn^2+^**	5 mM	102.7 ± 0.21^d^
**Fe^2+^**	5 mM	107.8 ± 0.52^e^
**Cu^2+^**	5 mM	108.5 ± 0.81^e^
**Co^2+^**	5 mM	102.3 ± 2.5^d^
**K^+^**	5 mM	109.9 ± 2.5^e^
**Zn^2+^**	5 mM	36 ± 2.2^f^
**Boric acid**	1 mM	98.3 ± 0.91^a^
**Potassium metaborate**	1 mM	100.0 ± 0.40^a^
**Sodium metaborate**	1 mM	98.6 ± 0.75^a^
**Sodium tetraborate**	1 mM	101.0 ± 2.5^a^
**Tween 40**	1% (w/v)	87.6 ± 1.0^b^
**Tween 60**	1% (w/v)	89.1 ± 2.5^b^
**Tween 80**	1% (w/v)	87.9 ± 0.97^b^
**Triton X-100**	1% (w/v)	100.0 ± 1.43^a^
**SDS**	1% (w/v)	47.7 ± 2.5^c^
**Sodium cholate**	1% (w/v)	106.0 ± 2.5^d^
**Saponin**	1% (w/v)	117.2 ± 1.9^d^
**Sodium carbonate**	10 mM	84.9 ± 2.3^e^
**Hydrogen peroxide**	0.1% (w/v)	49.1 ± 1.12^c^
**Sodium hypochlorite**	0.1% (w/v)	94.3 ± 0.90^a^
**Sodium perborate**	0.1% (w/v)	88.9 ± 1.34^b^
**Omo**	1% (w/v)	36.3 ± 1.04^c^
**Ariel**	1% (w/v)	43.4 ± 2.5^c^
**Perwoll**	1% (w/v)	69.2 ± 2.5^b^
**Boron**	1% (w/v)	94.8 ± 1.11^a^
**Pril**	1% (w/v)	93.6 ± 0.54^a^
**Pril dishwasher tablet**	1% (w/v)	107 ± 0.21^d^
**Finish dishwasher tablet**	1% (w/v)	92.8 ± 2.1^a^

a Residual activity of the untreated enzyme was set as 100%. Data are mean ± SD (*n* = 3). Different letters in the last column indicate significant differences at *p* < 0.05.

These additions do not affect the results, discussion, or conclusions of the manuscript. The corrected version of Table 2 is provided below.

