# Supplementary material for: Purification and Characterization of an Alkaline Lipase from Streptomyces sp. AU-153 and Evaluation of Its Detergent Compatibility
Source: ACS Omega. 2026 Jan 23;11(5):8470–8. doi: 10.1021/acsomega.5c11317 (PMC12902849; doi:10.1021/acsomega.5c11317)
Supplement: Supplementary file 1 [file ao5c11317_si_001.pdf]

Supporting Information

Purification and characterization of an alkaline lipase from *Streptomyces* sp. AU-153 and evaluation of its detergent compatibility

*Rukiye Boran Gulen[1], Aysel Ugur[*2], Nurdan Sarac[3]*

1Medical Laboratory Program, Department of Medical Services and Techniques, Vocational School of Health Service, Aksaray University, 68100 Aksaray, Turkey

*2Section of Medical Microbiology, Department of Basic Sciences, Faculty of Dentistry, Gazi University, 06500 Ankara, Turkey

3Department of Biology, Faculty of Science, Mugla Sitki Kocman University, 48000 Mugla, Turkey

*Section of Medical Microbiology, Department of Basic Sciences, Faculty of Dentistry, Gazi University, 06500 Ankara, Turkey

e-mail: ayselugur@hotmail.com

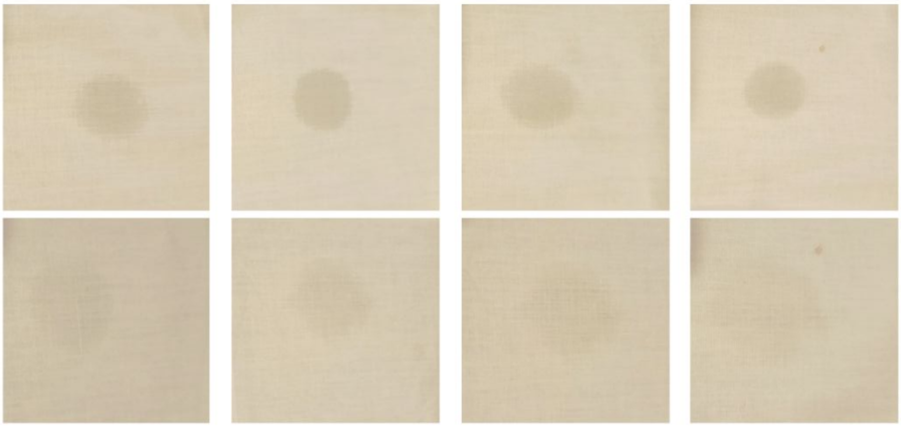

(i) (ii) (iii) (iv)

Figure S1. Sunflower oil stain removing property of the lipase of *Streptomyces* sp. AU-153 at 40 °C for 30 min. Pictures represent cloth pieces stained with oil (top line), and cloth pieces treated with tap water (i), lipase alone (ii), heat-inactivated detergent (iii), heat-inactivated detergent and enzyme (iv).
